# Smartphone-Based Remote Monitoring in Heart Failure With Reduced Ejection Fraction: Retrospective Cohort Study of Secondary Care Use and Costs

**DOI:** 10.2196/45611

**Published:** 2023-06-23

**Authors:** Sameer Zaman, Yorissa Padayachee, Moulesh Shah, Jack Samways, Alice Auton, Nicholas M Quaife, Mark Sweeney, James P Howard, Indira Tenorio, Patrik Bachtiger, Tahereh Kamalati, Punam A Pabari, Nick W F Linton, Jamil Mayet, Nicholas S Peters, Carys Barton, Graham D Cole, Carla M Plymen

**Affiliations:** 1 Imperial College London London United Kingdom; 2 Imperial College Healthcare National Health Service Trust London United Kingdom; 3 Imperial College Health Partners London United Kingdom

**Keywords:** heart failure, remote monitoring, smartphone care, telemonitoring, self-management, admission prevention, cohort study, hospitalization, noninvasive, smartphone, vital signs, diagnosis

## Abstract

**Background:**

Despite effective therapies, the economic burden of heart failure with reduced ejection fraction (HFrEF) is driven by frequent hospitalizations. Treatment optimization and admission avoidance rely on frequent symptom reviews and monitoring of vital signs. Remote monitoring (RM) aims to prevent admissions by facilitating early intervention, but the impact of noninvasive, smartphone-based RM of vital signs on secondary health care use and costs in the months after a new diagnosis of HFrEF is unknown.

**Objective:**

The purpose of this study is to conduct a secondary care health use and health-economic evaluation for patients with HFrEF using smartphone-based noninvasive RM and compare it with matched controls receiving usual care without RM.

**Methods:**

We conducted a retrospective study of 2 cohorts of newly diagnosed HFrEF patients, matched 1:1 for demographics, socioeconomic status, comorbidities, and HFrEF severity. They are (1) the RM group, with patients using the RM platform for >3 months and (2) the control group, with patients referred before RM was available who received usual heart failure care without RM. Emergency department (ED) attendance, hospital admissions, outpatient use, and the associated costs of this secondary care activity were extracted from the Discover data set for a 3-month period after diagnosis. Platform costs were added for the RM group. Secondary health care use and costs were analyzed using Kaplan-Meier event analysis and Cox proportional hazards modeling.

**Results:**

A total of 146 patients (mean age 63 years; 42/146, 29% female) were included (73 in each group). The groups were well-matched for all baseline characteristics except hypertension (*P*=.03). RM was associated with a lower hazard of ED attendance (hazard ratio [HR] 0.43; *P*=.02) and unplanned admissions (HR 0.26; *P*=.02). There were no differences in elective admissions (HR 1.03, *P*=.96) or outpatient use (HR 1.40; *P*=.18) between the 2 groups. These differences were sustained by a univariate model controlling for hypertension. Over a 3-month period, secondary health care costs were approximately 4-fold lower in the RM group than the control group, despite the additional cost of RM itself (mean cost per patient GBP £465, US $581 vs GBP £1850, US $2313, respectively; *P*=.04).

**Conclusions:**

This retrospective cohort study shows that smartphone-based RM of vital signs is feasible for HFrEF. This type of RM was associated with an approximately 2-fold reduction in ED attendance and a 4-fold reduction in emergency admissions over just 3 months after a new diagnosis with HFrEF. Costs were significantly lower in the RM group without increasing outpatient demand. This type of RM could be adjunctive to standard care to reduce admissions, enabling other resources to help patients unable to use RM.

## Introduction

Despite proven effective medical therapies, chronic heart failure with reduced ejection fraction (HFrEF) has a prognosis worse than most cancers [1] and accounts for a substantial health-economic burden [2]. A major driver of these high costs is frequent clinical decompensations requiring emergency department (ED) attendance and urgent hospital admissions [3]; reducing these is a primary target for remote monitoring (RM) interventions [4,5].

Community-based management by a heart failure specialist nurses (HFSNs) decreases hospitalizations but relies on high-frequency monitoring of vital signs and regular symptom review via serial face-to-face outpatient appointments [6]. In practice, these appointments are often too infrequent to capture rapid changes in a patient’s clinical status and allow early intervention. Patients may recognize their condition is deteriorating, but there is no systematic way of corroborating this with objective clinical data (eg, self-measurement of vital signs) or a convenient line of communication with clinicians who can intervene. This potentially misses a window of opportunity for early intervention, which may instead lead to ED attendance and admissions [7].

RM aims to optimize care and the implementation of guideline-directed medical therapy for HFrEF by providing a platform for the collection and transmission of clinical data at a higher frequency and more conveniently than serial face-to-face appointments. By leveraging clinical data submitted by patients remotely at their convenience, RM aims to facilitate timely community-based clinical intervention and avoid admissions to secondary care [4].

Existing research into RM in heart failure has yet to influence clinical guidelines due to a lack of consensus regarding which type of RM is most impactful [8,9]. Noninvasive RM of vital signs (rather than invasive data from implanted devices) has minimal risks to patients and is often cheaper than other strategies, so it can be applied to a greater proportion of heart failure patients [10]. The majority of noninvasive RM for HFrEF uses telephone-based strategies [11], which fail to harness the wide adoption of smartphone technology or meet the acceleration of demand for remote care brought about by the COVID-19 pandemic [12]. The impact of modern smartphone platforms that combine noninvasive RM of vital signs, messaging, and patient-focused e-learning is unknown. The risk of rehospitalization is highest after initial diagnosis, an opportune window for RM-based intervention [13]. Modifying what happens to patients during this period is often most relevant to patients and health systems considering whether RM is clinically and economically beneficial.

In this study, we present a clinical and economic evaluation of Luscii, a novel smartphone-based RM platform for HFrEF patients that have demonstrated feasibility for monitoring patients with other conditions [14,15]. Our primary objective was to conduct a secondary care health use and health-economic evaluation for patients with HFrEF using smartphone-based noninvasive RM and compare it with matched controls receiving usual care without RM.

## Methods

### Overview

We performed a retrospective clinical and health economic evaluation of a novel type of smartphone-based, noninvasive RM platform for patients with HFrEF. The platform combined a smartphone app with noninvasive self-measurement of blood pressure, pulse rate, and body mass that is transmitted to a cloud-based server. It also enabled self-reporting of heart failure symptoms, pill use, and messaging functionality to communicate with clinicians, together with a suite of tailored e-learning modules. We compared the impact of this type of RM to a matched group of controls receiving standard heart failure care without RM. We compared secondary health care use and associated costs over a 3-month period following a new diagnosis of HFrEF.

### Ethics Approval

This study was approved by the Imperial College Health Care audit and quality and improvement committee (CAR/077) and the North West London Sub-Data Research Access Group committee (sDRAG; ID-186). Study data were deidentified. Participants were not compensated for their involvement.

### Study Design

**Figure 1:**
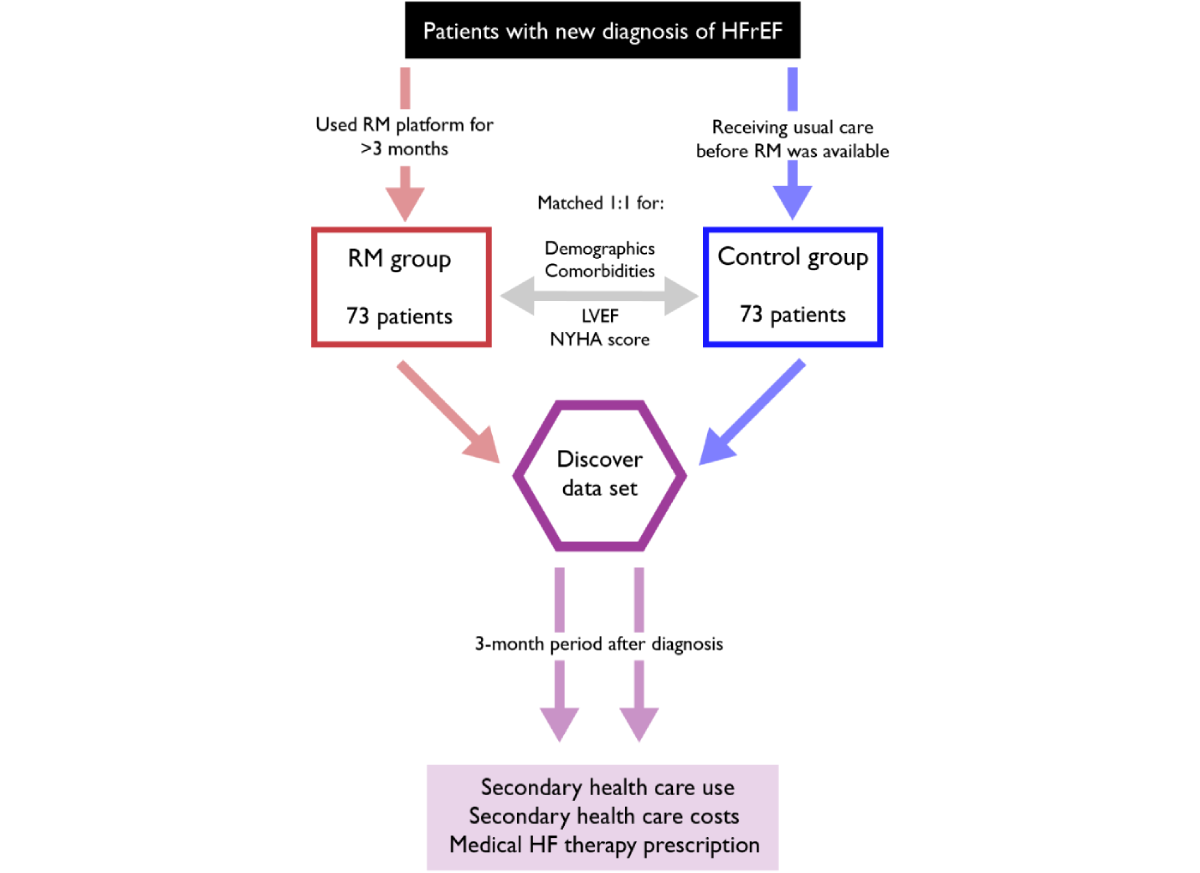
Study design. HF: heart failure; HFrEF: heart failure with reduced ejection fraction; LVEF: left ventricular ejection fraction; NYHA: New York Heart Association; RM: remote monitoring.

We performed a retrospective analysis of 2 cohorts (the RM group and the control group) with a new diagnosis of HFrEF, defined as heart failure and a left ventricular ejection fraction (LVEF) <50%. This cutoff combined patients in the “mildly reduced” and “reduced” ejection fraction groups as defined by international clinical guidelines [16]. The study design is summarized in Figure 1.

### Setting

We studied adult patients with a new diagnosis of HFrEF who were referred to our heart failure service in northwest London between October 2020 and November 2021.

### Participants

The RM group was defined by new HFrEF patients who were onboarded to the RM platform between April and November 2021 and used it for at least 3 months. The inclusion criteria included participants (1) with a new diagnosis of HFrEF (LVEF <50%), (2) who agreed to use the RM platform, (3) who used the platform for at least 3 months, and (4) with regular platform use (submitting at least 2 measurements per week).

The control group was defined as a group of consecutive new patients with HFrEF who were referred to our service before RM was available (October 2020 to March 2021). These patients were identified from our registry of heart failure referrals. These patients all received standard heart failure care without RM in accordance with international clinical guidelines [16]. From this group, a set of patients (the same number as in the RM group) were selected by matching to the RM group for a range of factors (see below). The ratio of RM to control patients was 1:1.

### Sample Size

The sample size was determined by the number of patients fulfilling the inclusion criteria above. The size of the control group was determined by the size of the RM group by 1:1 matching. We envisaged that the distribution of health care use and costs would be nonparametric. We calculated that detecting a change in the probability of ED attendance, hospital admission, and outpatient clinic attendance of 0.15 (with the null hypothesis assuming that there is no difference between groups, that is, probability of RM decreasing use=0.5), at an α of .05 and power of 80% would require at least 58 patients per group [17].

### The Remote Monitoring Intervention

#### Overview

The RM intervention in this study used the Luscii (Luscii Healthtech BV) platform. This is a commercially available smartphone-based RM platform. None of the authors of this study are employed by Luscii or were involved in the development of the platform. No employees of Luscii were involved in our analyses. The intervention combined 3 modules within a single smartphone app.

#### Measurements Module

Patients were given a digital sphygmomanometer, pulse rate monitor, and body mass scales, which were connected to the smartphone app via Bluetooth. Patients were prompted to submit measurements daily, with no upper limit on the number of allowable measurements. All previously submitted measurements were viewable by the patient and clinicians in graphical and tabulated formats. Patients could also complete optional questionnaires about heart failure symptoms, pill use, anxiety, and depression (Figure S1 in Multimedia Appendix 1 shows a screenshot).

#### Self-Care Module

e-Learning modules written by HFSNs in our department were uploaded to the Luscii app. These covered topics such as prognostic heart failure medication, information about different cardiac investigations, and device therapy (Figure S2 in Multimedia Appendix 1 shows a screenshot).

#### Messages Module

Patients had the option to add free-text comments to their measurements, which were sent to clinicians in the form of a message. In this module, clinicians (typically HFSNs) could respond to these messages or send new messages as unstructured free text. HFSNs were available to interact with patients using this module between 9 AM and 5 PM, Monday to Friday (Figure S3 in Multimedia Appendix 1 shows a screenshot).

### Standard of Care Received by the Control Group (Usual Care)

The heart failure care received by the control group was the usual standard provided to all patients before we started using RM. It consisted of a comprehensive clinical and biochemical assessment in accordance with international guidelines [16]. Upon diagnosis of new HFrEF, every patient is allocated a named consultant and HFSN. They have appointments face-to-face and by telephone to check their symptoms and up-titrate their medical therapy. At each face-to-face appointment, the patient’s blood pressure, pulse, and weight are measured. The frequency and interval of these appointments are individualized depending on the clinical condition, response to therapy, blood test results, and patient wishes. Typically, patients will initially be seen weekly after the diagnosis and then move to longer intervals as they enter the chronic stage of the condition. In terms of educational material, all patients are provided with leaflets about the condition and a pack of web-based resources compiled by heart failure charities. Heart failure specialist nurses proactively provide opportunistic education and information to patients and their caregivers with each clinical encounter.

### Variables for Cohort Matching

From the pool of patients with a new diagnosis of HFrEF referred to our heart failure service in the months before RM, a control group was selected using propensity matching in a 1:1 ratio with the RM group for the following categories: demographics (age, sex, and ethnicity); socioeconomic status as measured by indices of multiple deprivations (IMDs; income, employment, and education) [18]; medical comorbidities (ischemic heart disease, hypertension, atrial fibrillation, stroke, type 2 diabetes mellitus, chronic obstructive pulmonary disease, and chronic kidney disease); and heart failure severity as measured by LVEF and the New York Heart Association (NYHA) classification at the time of referral.

### Outcome Variables

The measured outcome variables were the number of ED attendances, unplanned hospital admissions, elective hospital admissions, and cardiology outpatient appointments for each group. The total costs associated with each type of hospital activity were also measured.

### Data Sources and Measurement

The variables for cohort matching, including demographics, cause of heart failure, LVEF, NYHA score, and medical comorbidities, were extracted from the electronic health record.

The outcome variables were extracted from the Discover data set [19]. The Discover data set was accessed via the Discover-NOW Health Data Research Hub for Real World Evidence through their data scientist specialists and information governance committee-approved analysts, hosted by Imperial College Health Partners.

Data were extracted for health care use over a 3-month time period, starting with either onboarding to the RM platform (for the RM group) or referral to our service (for the control group). The costs associated with each type of activity were also extracted. For the RM group, the platform costs were added to the health care use costs.

### Statistical Methods

The effectiveness of the propensity matching was confirmed using 2-tailed *t* tests for continuous variables and Fisher exact test for discrete variables (or nonparametric equivalents) to detect differences in baseline characteristics between the RM and control groups.

Differences in health care use and associated costs were analyzed using Wilcoxon rank sum tests. Kaplan-Meier analysis with Cox proportional hazards modeling was used to analyze the probability of avoiding ED attendance, unplanned admission, elective admission, and cardiology outpatient clinic use between the 2 groups. Univariate Cox proportional hazard modeling was performed for any baseline variables found to be significantly different between the 2 groups.

## Results

### Overview

A total of 146 patients (42/146, 29% female, mean age 63.8 years) with HFrEF were included. The RM group included 73 patients with a new diagnosis of HFrEF who were onboarded to the RM platform and used it for at least 3 months. The control group included 73 patients with a new diagnosis of HFrEF from the period just before RM was available, matched to the RM group for age, sex, ethnicity, IMD, medical comorbidities, LVEF, and NYHA score at baseline. The baseline characteristics of the 2 groups are shown in Table 1. The groups were well-matched for demographics, IMD, heart failure severity, and comorbidities except for hypertension (RM group 27/73, 36%; control group 41/73, 55%; *P*=.03).

**Table 1:** Baseline characteristics. Baseline characteristics in the RM and matched control groups.

Baseline characteristic			RM^a^ group (n=73)	Control group (n=73)		*P* value
**Demographics**						
	Age (years), mean (SD)	63.0 (13.2)		64.5 (13.0)	.48	
	Female, n (%)	21 (29)		21 (29)	>.99	
	IMD^b^ decile, mean (SD)	3.40 (2.18)		3.87 (2.26)	.22	
**Ethnicity, n (%)**						
	White	40 (55)		42 (58)	.87	
	Black	13 (18)		11 (15)	.82	
	Asian	7 (10)		7 (10)	>.99	
	Mixed	5 (7)		7 (10)	.76	
	Other	8 (11)		11 (15)	.62	
**Medical comorbidities, n (%)**						
	Ischemic heart disease	24 (32)		23 (31)	>.99	
	Atrial fibrillation	22 (29)		24 (32)	.86	
	Hypertension	27 (36)		41 (55)	.03	
	Stroke	5 (<7)		7 (9)	.76	
	Type 2 diabetes mellitus	13 (17)		24 (32)	.06	
	COPD^c^	10 (13)		13 (17)	.65	
	Chronic kidney disease	8 (11)		11 (15)	.62	
**Heart failure parameters, %**						
	LVEF^d^, mean (SD)	33 (10)		32 (9)	.53	
**NYHA^e^ classification, n (%)**						
	I	11 (15)		14 (19)	.66	
	II	36 (49)		33 (45)	.74	
	III	22 (30)		25 (34)	.72	
	IV	4 (5)		1 (1)	.37	

^a^RM: remote monitoring.

^b^IMD: indices of multiple deprivation.

^c^COPD: chronic obstructive pulmonary disease.

^d^LVEH: left ventricular ejection fraction.

^e^NYHA: New York Heart Association.

### Secondary Health Care Use

Over the 3-month follow-up period, there were significantly fewer ED attendances in the RM group compared to the control group (16 vs 46, *P*=.01). The RM group also had fewer unplanned hospital admissions (4 vs 21, *P*=.01). There was no difference in elective (planned) hospital admissions (6 vs 5, *P*=.99) between the 2 groups. The RM group had a trend toward more cardiology outpatient use than the control group, but this difference did not reach statistical significance (77 vs 48, *P*=.10; Table 2).

**Figure 2:**
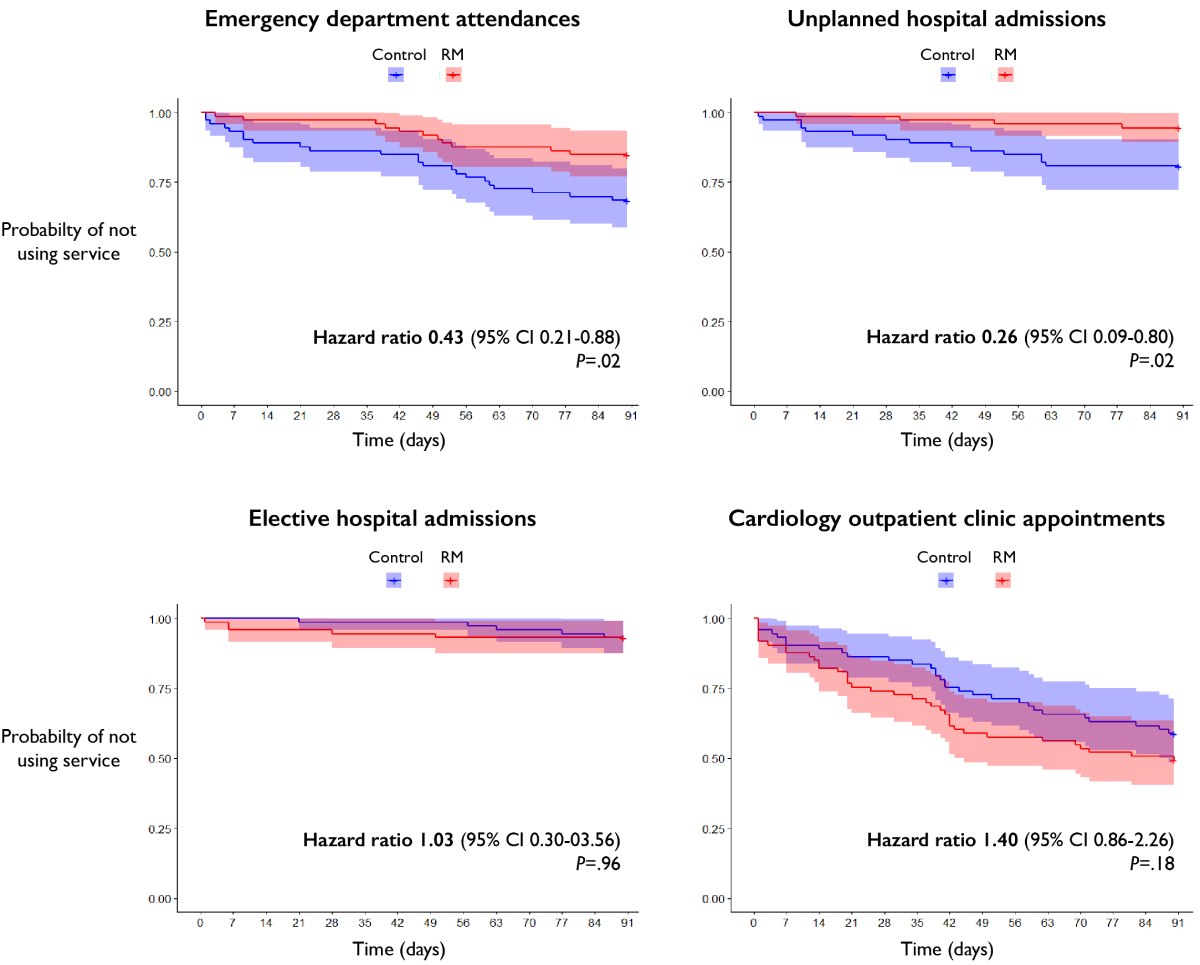
Event analysis for secondary health care use. Kaplan-Meier curves comparing the probability of not using different secondary health care services between RM and control groups. RM: remote monitoring.

Kaplan-Meier analyses (Figure 2) and Cox proportional hazard modeling (Table 3) showed that patients in the RM group had a significantly lower chance of attending an ED (unadjusted hazard ratio [HR] 0.43; 95% CI 0.21-0.88; *P*=.02) and having an unplanned hospital admission (unadjusted HR 0.26; 95% CI 0.09-0.80; *P*=.02). These findings were sustained by a univariate model that adjusted for hypertension (the one baseline characteristic that was unequal between the 2 groups): ED attendances (adjusted HR 0.43; 95% CI 0.21-0.89; *P*=.02) and unplanned hospital admissions (adjusted HR 0.29; 95% CI 0.09-0.89; *P*=.03).

**Table 2:** Secondary health care use and costs: The amount of secondary health care use and associated costs (categorized by type of encounter) by patients in the RM and control groups during a 3-month follow-up period. Use values are total counts of the number of events that took place in each category for each group. Cost values are the total spend in each category for each group (ie, not per patient). *P* values were calculated from Wilcoxon rank sum tests between median values for each group.

			RM^a^ group (n=73)		Control group (n=73)		*P* value
**Secondary health care use (total number of events in 3 months), n**							
	Emergency department attendances	16		46		.01	
	Unplanned admissions	4		21		.01	
	Elective admissions	6		5		.99	
	Cardiology outpatient attendances	77		48		.10	
**Secondary health care costs (GBP £, total)^b^**							
	Emergency department cost	2562		6673		.04	
	Unplanned admissions cost	11,321		108,906		.02	
	Elective admissions cost	5053		13,175		>.99	
	Cardiology outpatient cost	8827		6320		.07	

^a^RM: remote monitoring.

^b^A currency exchange rate of GBP £1=US $1.25 is applicable.

**Table 3:** Cox proportional hazard modeling for secondary health care use: hazard ratios (unadjusted and adjusted for hypertension) for different types of secondary health care use during a 3-month period. Hazard is calculated for the remote monitoring group with respect to the control group.

Type of health care use	Unadjusted		Adjusted for hypertension	
	HR^a^ (95% CI)	*P* value	HR (95% CI)	*P* value
Emergency department attendance	0.43 (0.21-0.88)	.02	0.43 (0.21-0.89)	.02
Emergency admission	0.26 (0.09-0.80)	.02	0.29 (0.09-0.89)	.03
Elective admission	1.03 (0.30-3.56)	.96	1.17 (0.33-4.16)	.80
Cardiology outpatient	1.40 (0.86-2.26)	.18	1.34 (0.82-2.19)	.24

^a^HR: hazard ratio.

### Secondary Health Care Costs

The secondary health care costs were largely in line with the amount of use. Over 3 months, the RM group had significantly lower cumulative ED attendance costs (total GBP £2562, US $3214.07 vs GBP £6673, US $8371.39; *P*=.04) and cumulative emergency hospital admissions (total GBP £11,321, US $14,202.38 vs GBP £108,906, US $136,624.32; *P*=.02). Although the RM group had lower total costs associated with elective hospital admissions and higher total outpatient costs than the control group, these differences did not reach statistical significance (Table 2).

When all 4 categories of secondary health care use over the 3-month period were considered, including (for the RM group) the cost of RM over this period, the RM group had a significantly lower mean cost per patient (GBP £465, US $583.35 vs GBP £1850, US $2320.85; *P*=.04).

## Discussion

### Primary Findings

The key findings of this study are that in the 3 months after HFrEF diagnosis, smartphone-based noninvasive RM was associated with a reduced hazard of ED attendance and unplanned hospital admissions by 57% and 74%, respectively, which was not explained by an increase in elective admissions or outpatient use. Over a 3-month period, the RM group had overall lower secondary health care costs than the control group, even after accounting for the cost of RM.

### Comparison With Previous Studies

Our results add evidence in support of smartphone-based noninvasive RM of vital signs in patients with HFrEF. To date, this is the largest study investigating the impact of a smartphone-based RM platform combining vital signs monitoring, patient-focused e-learning, and instant messaging with clinicians in this population. Our findings build on previous studies, which found that this type of RM was feasible and beneficial but was limited by low numbers [20,21] or the lack of matched controls [22]. Our findings also agree with a meta-analysis of telephone-based RM (which has been much more extensively researched for heart failure) that found it to reduce hospitalizations [23].

Many randomized trials of telephone-based RM such as BEAT-HF (Better Effectiveness After Transition–Heart Failure) and Tele-HF are limited by low adherence and high dropout (almost half at 6 months) [24,25]. By contrast, there was a very low dropout rate in our study; 87 patients were onboarded altogether, of whom 11 were excluded because they had under 3 months’ platform use at the time of analyses, and 3% (3/87) dropped out (2 stopped using and 1 died). This is in line with previous work for smartphone-based RM platforms [13]. There are a number of possible explanations. First, a smartphone app-based platform allows the flexibility of asynchronous communications, meaning patients can use it at a time convenient for them rather than a scheduled phone call [26]. Second, this type of RM enables vital sign and symptom self-reporting more frequently than face-to-face outpatient appointments but is not as intrusive as daily telephone calls; this data collection and trend analysis could aid therapy optimization and intercept clinical decompensation without burdening patients with fixed activities that may risk disengagement [7]. Third, we studied a self-selecting group who agreed to have RM and use the smartphone platform. This group was younger (mean age 63 years) than the average HFrEF patient, and almost all patients were able to use the technology without dropping out. This is similar to the average age of the RM cohort in another study [13], and it highlights the importance of the careful selection of the most appropriate patients for this type of RM: those who can use the technology and therefore stand to benefit from it [7].

### Smartphone-Based RM Combines Multiple Interventions Into a Single Platform

The RM technology used in this study packages together monitoring, education, self-care, and messaging features in a single smartphone app interface. It is difficult to tease apart which of these were most responsible for the reduction in acute secondary care use and costs that we observed. Simply having a direct line of communication with HFSNs may enable rapid, ad hoc decision-making such as temporary up-titration of diuretic doses, which could prevent admission. An instant messaging intervention has previously been found to reduce symptoms and improve quality of life [27]. Similarly, access to a nurse-led heart failure education program could increase patient activation, understanding, and self-management sufficiently to reduce the need for other health care services [28]. It is more likely that the additive benefit of all these features rather than any single one and the convenience of a “one-stop-shop” RM user interface are responsible for the differences between RM and control groups. Further follow-up is required to determine whether these differences are sustained in the long term.

### Secondary Health Care Economic Impact of RM

A key finding of this study is that the cost savings associated with lower ED attendance and unplanned hospital admissions in the RM group were not offset by higher use of elective and outpatient secondary care. This adds depth to previous findings that RM reduced hospitalizations but could not say whether use (and costs) were simply diverted to other parts of the health system [13,21,22]. Previous economic analyses of RM in heart failure have not accounted for RM platform costs, but this is a very important factor for decision makers considering the cost-effectiveness of RM. In our study, we added the RM platform costs to the secondary health care costs and found that the cost savings were sustained. We found that the largest difference in costs between the RM and control groups was for unplanned admissions rather than ED attendances. This might be because patients in the RM group had a shorter length of hospital stay than those in the control group due to being more optimized to start with.

### Impact on Other Medical Comorbidities

It is likely that the benefits of noninvasive vital sign RM extend beyond just the optimization of heart failure. Blood pressure, body mass, and heart rate are key parameters reflecting the optimization of other conditions such as hypertension, chronic kidney disease, obesity, and atrial fibrillation [26]. Optimizing these parameters is known to reduce the risk of ischemic heart disease and stroke [29]. It is intuitive to conclude that optimizing one condition using this method of RM has added benefits to patients’ comorbidities (which, as we observed, were highly prevalent in both groups in this study). Furthermore, since smartphone-based RM platforms also enable the delivery of educational material, the resulting increase in patient activation and self-management is likely to have far-reaching benefits beyond just heart failure care. Smartphone-based vital sign RM could be an effective holistic intervention to optimize care across a range of syndromes for multimorbid patients carefully selected based on their comorbidities and ability to use the technology effectively [7,26].

### Hypotheses for Improved Clinical Outcomes in the RM Group

There are a number of possible reasons why better clinical outcomes were observed in the RM group in this study. First, the RM platform may enable earlier recognition of clinical deterioration by monitoring physiological parameters (heart rate, blood pressure, weight, and symptoms) more frequently than is possible with traditional models of care using face-to-face appointments [30]. Second, previous research has shown that nurses have twice as much activity with RM patients as controls [31]. This closer attention may enable more aggressive up-titration of prognostic medical therapy (eg, on a daily rather than weekly basis), leading to fewer hospital admissions. Patients in the control group would typically have to wait for their next face-to-face appointment or telephone consultation or seek emergency medical care if their symptoms or measurements worsened. Third, the RM platform provided patients with more opportunities to engage in their health care both passively (when inputting their parameters) and actively (when engaging with specialists via the instant messaging platform or undertaking the learning modules). This may lead to increased medication adherence, as seen in previous studies [32]. Finally, the RM platform might make information previously available to patients in the form of leaflets more accessible to patients. Nurse-led education is known to reduce readmissions and improve quality of life, which may be linked to the clinical outcomes observed in our study [33].

### Limitations

The primary limitation of this study is the retrospective, nonrandomized design. As a retrospective cohort study, this study may have selection bias compared to a randomized controlled trial. We accounted for this by matching the RM group with a control group matched for a wide range of demographic, socioeconomic, and clinical features. This design is novel compared to other studies investigating noninvasive RM of vital signs in HF [13,21,22]. Our matching process was effective (Table 1) for all categories except hypertension. We further accounted for this with a univariate Cox proportional hazards model that controlled for hypertension (Table 3). Importantly, since socioeconomic status and education level are linked to smartphone use, we controlled for the 7 IMDs: income, employment, education, health, crime, housing, and living environment. Both groups were well-matched for IMDs (*P*=.22); therefore, we assert that these factors do not confound our results. Although there may be other minor confounders, these are likely to be equally distributed between both groups. Our fastidious approach to cohort matching may explain the positive findings in our study, despite it not being a randomized design. We believe this is an important contribution to the literature to stimulate more rapid adoption of these types of technologies so that patients can start benefiting from them sooner. We strongly recommend the formal evaluation of the long-term efficacy of this type of RM by means of a randomized controlled trial.

It is possible that the patients in the RM group were more proactive and engaged with their care than the control group, even before they used the RM. This may in part explain our results. However, if this were the case, it would typically be driven by previous experience using digital technologies and smartphone ownership. The primary determinants of smartphone ownership are income, education, and socioeconomic category, which we controlled. Therefore, it is unlikely that this was a significant source of bias that explains our results. Our aim in sharing the results of this study is to stimulate further adoption of this type of technology in clinical practice. We recommend future researchers verify our findings via large randomized controlled trials.

We used propensity score matching to match patients between the RM and control groups in a 1:1 ratio. This method of matching may introduce minor bias to the results of conventional Cox regression modeling due to a lack of independence between the 2 groups [34]. In the absence of consensus on this topic, it is statistically more conservative to assume the groups are independent, and our analyses support this. We encourage future researchers to account for time-dependent exposure by adjusting propensity scores for this to potentially enable unbiased estimates [35].

The study duration was only 3 months. This follow-up length is similar to other studies [13,22], but ours includes more patients and a matched control group. We focused on patients with a new diagnosis of HFrEF. Registry data show that the risk of decompensation—and therefore the largest window for RM intervention—is in the weeks after diagnosis [36]. 3 months is therefore an appropriate timeframe to evaluate the impact of RM on the optimization of care as measured by health care use, costs, and prescribing.

The patients in both cohorts in this study had an average age younger than that of all-comers with HFrEF (64 years). Although this does not confound our results (because the groups are matched), it reflects the fact that older patients (in general) did not opt for this RM strategy. This may be because they are unwilling or unable to use the technology or do not own a smartphone, as has been reported by previous researchers [21]. It is important that RM technologies do not worsen health inequalities, in particular for groups that may not have access to smart devices or reliable internet connections [37]. As a result, we recommend that RM be viewed as a supplement to, not a replacement for, standard clinical care. A major contribution of RM technologies may be to optimize management remotely for those for whom it is possible and desirable, enabling redistribution of resources to enhance standard care for those who are unwilling or unable to have RM [7].

### Conclusions

This study demonstrates that smartphone-based noninvasive RM of vital signs combined with a messaging platform and e-learning is feasible for patients with HFrEF. In the 3-month period after diagnosis, RM was associated with significantly lower ED attendance and unplanned hospital admissions without placing extra demand on elective care or outpatient clinics. The secondary health care costs of the RM group were significantly lower than standard care without RM, even after accounting for the costs of RM itself.

Based on these findings, RM has significant benefits for patients and health systems in the early period after a diagnosis of HFrEF. Noninvasive RM should be viewed as an adjunct to standard care to reduce admissions and enable other complementary resources to be directed toward patients who are unable to use RM.

## Appendix

Multimedia Appendix 1 Screenshots of the remote monitoring intervention smartphone app.

## Abbreviations

BEAT-HF: Better Effectiveness After Transition–Heart Failure
ED: emergency department
HFrEF: heart failure with reduced ejection fraction
HFSN: heart failure specialist nurse
HR: hazard ratio
IMD: index of multiple deprivation
LVEF: left ventricular ejection fraction
NYHA: New York Heart Association
RM: remote monitoring

## Data Availability

The data sets generated during and/or analyzed during this study are available from the corresponding author on reasonable request.
