# Supplementary material for: Smartphone-Based Remote Monitoring in Heart Failure With Reduced Ejection Fraction: Retrospective Cohort Study of Secondary Care Use and Costs
Source: JMIR Cardio. 2023 Jun 23;7:e45611. doi: 10.2196/45611 (PMC10334716; doi:10.2196/45611)
Supplement: Multimedia Appendix 1 [file cardio_v7i1e45611_app1.docx]

**Multimedia Appendix 1**

**Screenshots of the remote monitoring intervention smartphone app**

**
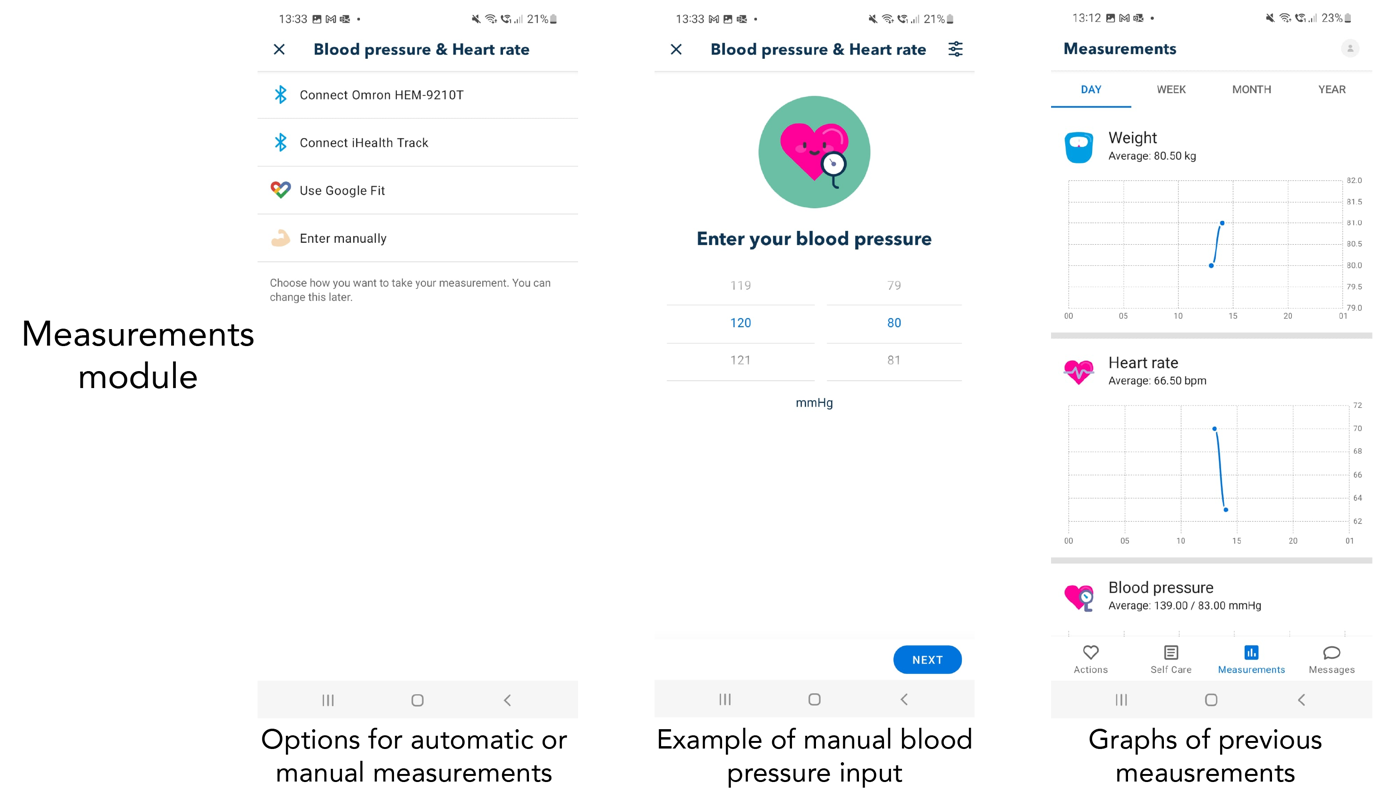
**

Figure S1. Screenshots of the measurements module in the Luscii smartphone app.

**
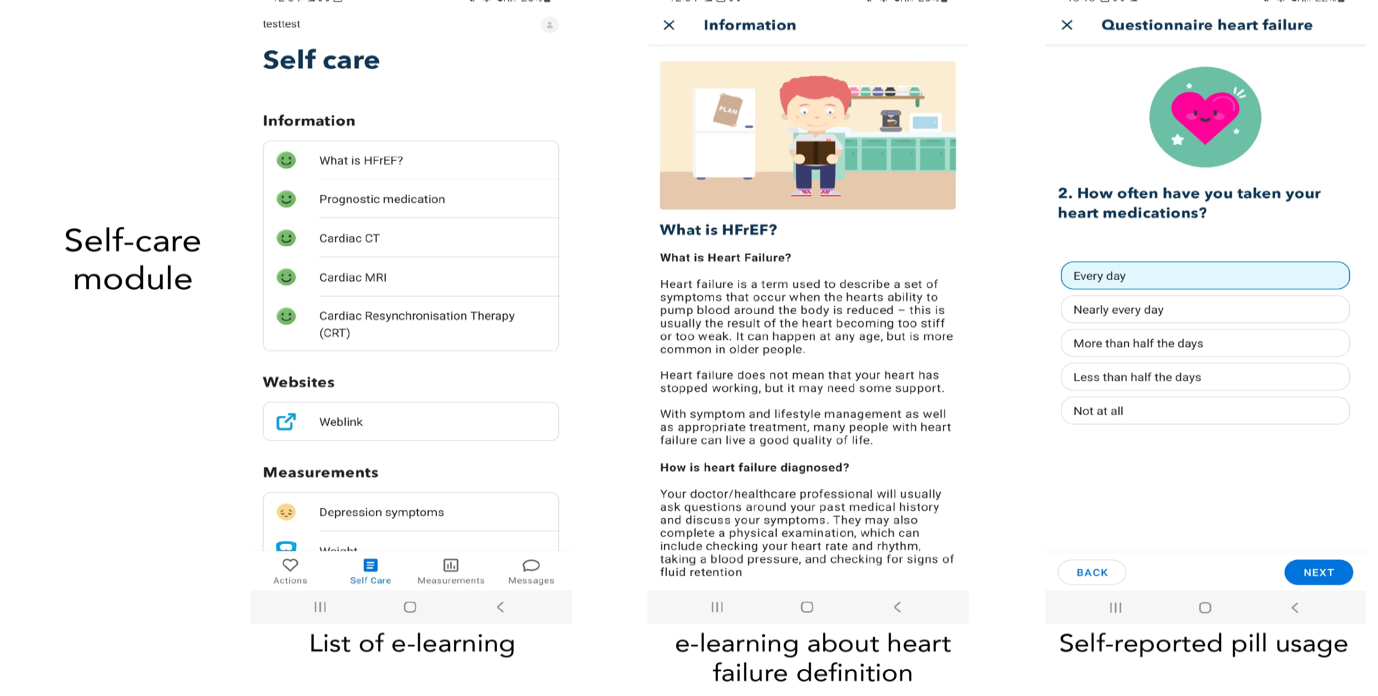
**

Figure S2. Screenshots of the self-care module in the Luscii smartphone app.


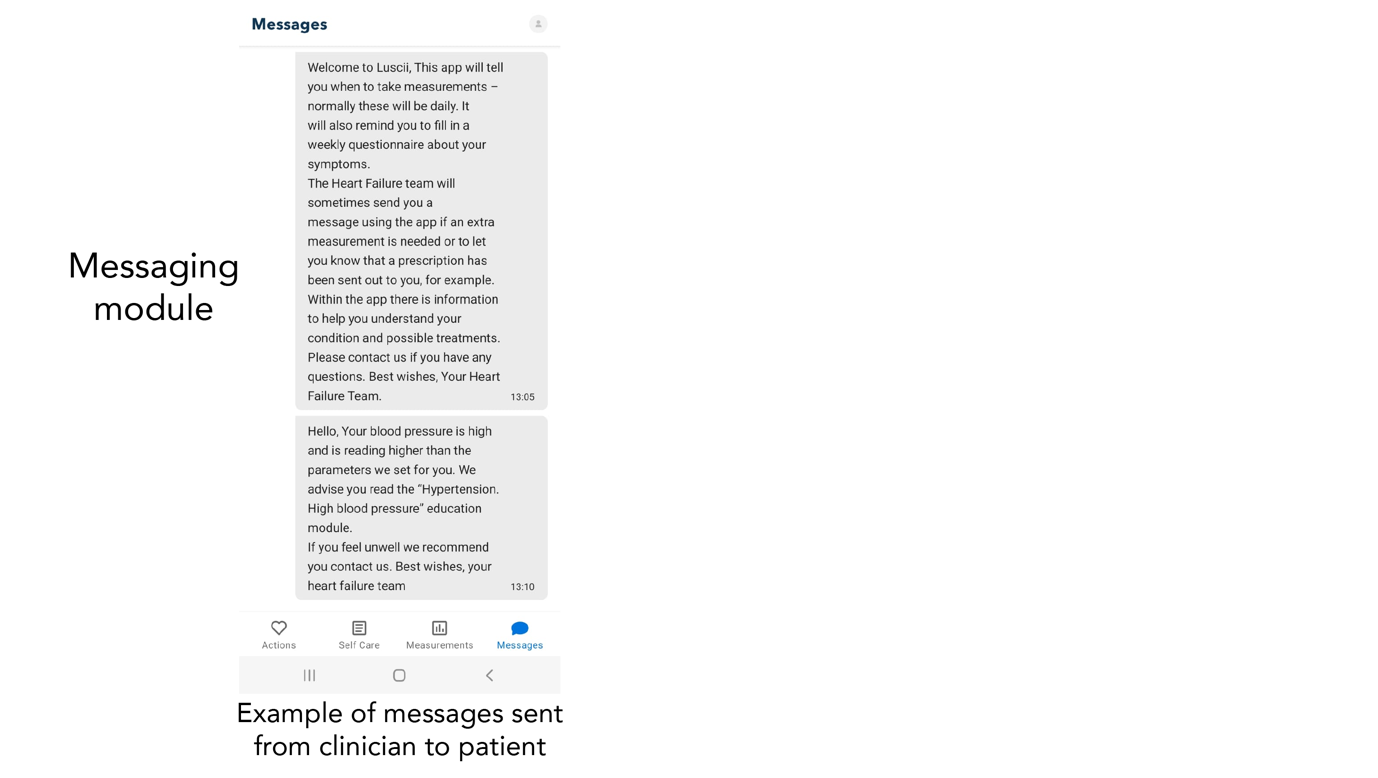
The self-care module consisted of text and graphical media covering a range of topics and self-test questions related to heart failure patient perspectives including “what is heart failure?”, “living with heart failure”, “how the medicines work”, “sick day rules” etc. There was no specific theoretical framework applied, but the basis was informal feedback from patients and carers who suggested topics and themes that they wanted to know more about. The material was developed and written by heart failure specialist nurses with extensive first-hand experience of answering queries from patients living with heart failure, rather than generic medical writers who create most other content available on these topics. The primary novelty of the self-care module was its completeness, accessibility and ease of availability, providing a one-stop-shop for patient-facing HF information in a single app resource.

Figure S3. Screenshot of the messages module in the Luscii smartphone app.
